# Functional and biochemical characteristics of urinary bladder muscarinic receptors in long-term alloxan diabetic rats

**DOI:** 10.1590/S1679-45082015AO3233

**Published:** 2015

**Authors:** Jeová Nina Rocha

**Affiliations:** 1 Faculdade de Medicina de Ribeirão Preto, Universidade de São Paulo, Ribeirão Preto, SP, Brazil.

**Keywords:** Urinary bladder/metabolism, Alloxan/adverse effects, *Diabetes mellitus*, experimental, Receptors, muscarinic, Urination/drug effects, Rats, Wistar

## Abstract

**Objective:**

To re-examine the function of the urinary bladder *in vivo* as well as to determine the functional and biochemical characteristics of bladder muscarinic receptors in long-term alloxan-induced diabetes rats.

**Methods:**

Two-month-old male Wistar rats were injected with alloxan and the animals showing blood glucose levels >300mg/dL together with age-paired untreated animals were kept for 11 months. Body weight, bladder weight, blood glucose, and urinary volume over a period of 24 hours were determined in both groups of animals. A voiding cystometry in conscious control and diabetic rats was performed to determine maximal micturition pressure, micturition contraction interval and duration as well as voided and post-voiding residual volume. In addition, concentration-response curves for bethanechol in isolated bladder strips, as well as [^3^H]-N methyl-scopolamine binding site characteristics in bladder homogenates were determined.

**Results:**

Mean bladder weight was 162.5±21.2mg *versus* 290±37.9mg in control and treated animals, respectively (p<0.05). Micturition contraction amplitude (34.6±4.7mmHg *versus* 49.6±2.5mmHg), duration (14.5±1.7 seconds* versus* 23.33±4.6 seconds) and interval (87.5±17.02 seconds* versus* 281.11±20.24 seconds) were significantly greater in alloxan diabetic rats. Voided urine volume per micturition contraction was also significantly higher in diabetic animals. However the post-voiding residual volume was not statistically different. Bethanechol potency (EC50 3µM *versus* 5µM) and maximal effect (31.2±5.9g/g *versus* 36.1±6.8g/g) in isolated bladder strips as well as number (169±4fmol/mg *versus* 176±3fmol/mg protein) and affinity (0.69±0.1nM *versus* 0.57±0.1nM) of bladder muscarinic receptors were also not statistically different.

**Conclusion:**

Bladder function *in vivo* is altered in chronic alloxan-induced diabetes rats without changes in functional and biochemical characteristics of bladder muscarinic receptors.

## INTRODUCTION

Following the initial description of six diabetic patients presenting with paralysis of the urinary bladder by Jordan et al.^1^ accumulated evidence indicate that chronic diabetic patients frequently exhibit symptoms of urinary bladder dysfunction.^2^ Diabetic bladder dysfunction symptoms, termed diabetic cystopathy when associated with peripheral neuropathy,^3^ include micturition hyporeflexia, higher bladder capacity, urinary retention and even complete bladder atony.

The effects of chronic diabetes on urinary bladder function were studied in different animal models of diabetes. Although most studies showed an increase in bladder compliance and in the threshold volume required to elicit micturition contractions, conflicting results regarding the contractile function of the detrusor muscle and its nervous control in diabetic animals were described. For instance, cystometry (CMG) studies under isovolumetric conditions in 4 and 6 months diabetic Big Blue (BB) rats^4^ showed an increase in threshold volume and amplitude of bladder contractions. In contrast, in 3 and 6 months alloxan-induced diabetes rats no differences were observed in the amplitude of bladder contractions under isovolumetric conditions.^5,6^Similarly, CMG studies under voiding conditions, in anesthetized 7-9-week-old streptozotocin (STZ) induced diabetes rats, showed no differences in the amplitude of micturition contractions.^7,8^ However, in 8-11-week old STZ-induced diabetic rats, a voiding CMG study under non-anesthetized conditions showed that the amplitude of micturition contractions was greatly increased.^9^ In contrast, in a recent study using 10- and 46-week-old Goto-Kakizaki diabetic rats, it was observed that the amplitude of micturition contractions was significantly diminished.^10^

Conflicting observations were also described regarding detrusor contractile response to nerve stimulation or to muscarinic agonists; for instance, detrusor contractions elicited by muscarinic agonists were reported as increased,^11-13^ decreased^14^ or unchanged in diabetic animals.^15^

## OBJECTIVE

To re-examine the function of the urinary bladder *in vivo* as well as to determine the functional and biochemical characteristics of bladder muscarinic receptors in rats presenting long-term alloxan-induced diabetes.

## METHODS

Experimental procedures were approved by the Animal Ethics Committee, of the *Faculdade de Medicina de Ribeirão Preto da Universidade de São Paulo* (Protocol #051/2011). All experiments were conducted at the Neurourology Laboratory of the *Faculdade de Medicina de Ribeirão Preto*.

Diabetes was induced in 2-month old male Wistar rats by a single intravenous injection of alloxan (40mg/kg, diluted in 0.9% saline solution); two days after alloxan administration, non-fasted pooled blood samples were obtained from the tail for determination of whole blood glucose levels (glucose analyzer Beckman, Brea, California, United States). The animals with blood glucose levels ≥300mg/dL were considered diabetic and included in the study. Untreated age-paired rats were included as controls. Both groups of animals were kept for 11 months under similar housing conditions (room temperature 22±2°C, 12:12-hour light-dark cycle), with *ad libitum* access to food and water.

Eleven months after alloxan injection, non-fasted pooled blood samples were obtained from the tail from both groups to determine glucose levels in whole blood. Thereafter, control and diabetic animals were placed in metabolic cages to determine their 24-hour urinary output. The metabolic cages had a special net that allowed urine to pass separately from feces. After the metabolic cage study, four control and five diabetic rats were anaesthetized with sulfuric ether, had their ventral abdominal wall shaved and cleaned with betadine and their bladders exposed via a lower midline abdominal incision. One end of a polyethylene (PE-50) catheter with a flared tip to make a small collar was introduced in the bladder at the dome and secured with a ligature. The other end of the catheter was tunneled through the subcutaneous space and externalized at the lumbar region. Animals were then placed in special restraining cages designed to collect the urine voided with each micturition contraction. A continuous voiding CMG was performed after the rats had recovered completely from anesthesia. For this procedure an urinary catheter was connected via a three-way stopcock to a pressure transducer (World Precision Instruments Inc., Sarasota, United States) and to an infusion pump (KD Scientific Infusion Pump, Model 780.200, Series number 201755, United States). The pressure transducer was placed at the level of the pubic bone of the animal and saline solution (at room temperature) was continuously infused (flow rate 0.2mL/min) to elicit micturition contractions. Intravesical pressure (IVP) was continuously recorded in thermal paper using a multichannel polygraph (Narco BioSystems MK III, Houston, Texas, United States). After 20 to 30 minutes equilibrium period, a 30-minute CMG was recorded for analysis. The following parameters of the CMG were analyzed: maximal micturition pressure (IVP pressure developed during micturition contractions); micturition interval (time interval between return of IVP to baseline after a micturition contraction and the beginning of the next micturition contraction, that is, the time point at which the IVP showed a rapid increase); duration of micturition contractions, time lapsed from the beginning of the micturition contraction (see above) up to the return to baseline of the IVP. After recording the CMG for 30 minutes the infusion was stopped and the bladder was emptied. The infusion was then reinitiated and continued until the emission of urine accompanying the first micturition contraction started. The volume of voided urine was then measured and used to calculate the post-voiding residual volume using the formula: volume of infused saline minus the voided volume.

### *In vitro* experiments

After the metabolic cage study, other four control and four diabetic rats were anaesthetized with sulfuric ether had their bladders exposed via a lower midline abdominal incision and removed. The bladders were placed in a Petri dish containing a modified Krebs solution (in mM: NaCl 119.4; NaHCO_3_ 25.00; KCl 4.7; MgCl_2 _1.31; CaCl_2 _2.5; KH_2_PO_4 _1.17; glucose 11.1) and after removing adherent tissues longitudinal a strip (15-mm long x 3-mm wide) of the bladder wall was excised and mounted for recording of isometric contractions in a 10mL organ bath, containing the same Krebs solution kept at 37°C and continuously bubbled with 95% O_2_/5% CO_2_. The detrusor strip, under 1g basal tension, was allowed to stabilize for 60 minutes, changing the Krebs solution every 15 minutes. Following this equilibrium period, full cumulative concentration-response curves for the muscarinic agonist bethanechol were designed. After the experiment bladder strips were removed, blotted on filter paper and weighed. The weight of the strips was used to normalize the contractile force.

### Biochemical determinations

After the metabolic cage study, six animals, three control and three diabetic, were anaesthetized with sulfuric ether, had their bladders removed, cut into small fragments and homogenized immediately in ice-cold HEPES Na^+^/Mg^+^, pH 7.4 (NaCl=100mM, MgCl_2_=10mM and HEPES=20mM) with a Brinkman Polytron set at speed 6 (twelve 5-second bursts at 15-second intervals). The homogenate was filtered through nylon mesh and centrifuged at 13.000rpm for 20 minutes. The supernatant fraction was discarded and the pellet was re-dissolved in HEPES buffer and diluted to a final protein concentration of 1.0mg/m. Protein concentration was determined by the method of Lowry et al.^16^ using bovine serum albumin as standard. Duplicate samples of 0.5mL of the homogenate (corresponding to 0.5mg protein) were incubated at 32°C, in a Dubnoff shaker for 45 minutes, with several concentrations of [^3^H]-N-methyl-scopolamine ([^3^H]-NMS, 0.1 to 10nM) in the absence and presence of a 100 times excess of non-labeled scopolamine. Incubation was terminated by adding ice-cold HEPES buffer and rapid filtration under vacuum through GF/B glass fiber filters. Filters were rinsed twice with ice-cold HEPES buffer and transferred to scintillation vials containing 8mL of Aquasol (NEN, Boston Massachusetts, United States). After vigorous shaking, the filters were left for 24 hours in the dark to extract the radioactivity and counted in a Liquid Scintillation counter (Packard 1500, California, United States) for 2 minutes. Specific binding was defined as the difference of radioactivity in the presence and absence of scopolamine for each concentration of [^3^H]-NMS. Kd and Bmax of specific binding expressed per mg of protein was calculated using the linear regression plot of Scatchard.

### Statistical analysis

All values of CMG parameters and of maximal tension of bladder strips are expressed as mean±SEM. The concentration of bethanechol required to cause contractions of a magnitude equal to 50% of the maximum (EC_50_) for each strip was calculated as the geometrical mean. Statistical significance of differences between control and diabetic rats was determined using the Student’s *t* test. A value of p<0.05 was considered as statistically significant.

## RESULTS

Blood glucose levels in the alloxan-induced diabetes animals were significantly higher than in the control animals (p<0.05) (Table 1). Both groups of animals exhibited an increase in body weight during the 11-month period; however, alloxan-induced diabetes rats gained significantly less weight. Diabetic rats also showed significantly higher bladder weight and 24 hours urinary output (Table 1).

**Table 1:** Effect of the *diabetes mellitus* induced by alloxan on the body weight of the rat, bladder weight, blood glucose concentration and diuresis of 24 hours

	Control (n=8)	Diabetic (n=9)
Body weight, g		
Initial g	150.0±5.0	160.0±20.0
Final g	520.0±30.8	330.0±10.0*
Bladder weight, mg	162.5±21.2	290.0±37.9*
Blood glucose, mg/dL	118.0±17.1	536.0±25.9*
Diuresis, mL/24hours	20.2±3.7	103.0±7.85*

*Statistically different from control (p<0.05).

### Continuous voiding cystometrogram

**Figure 1:**
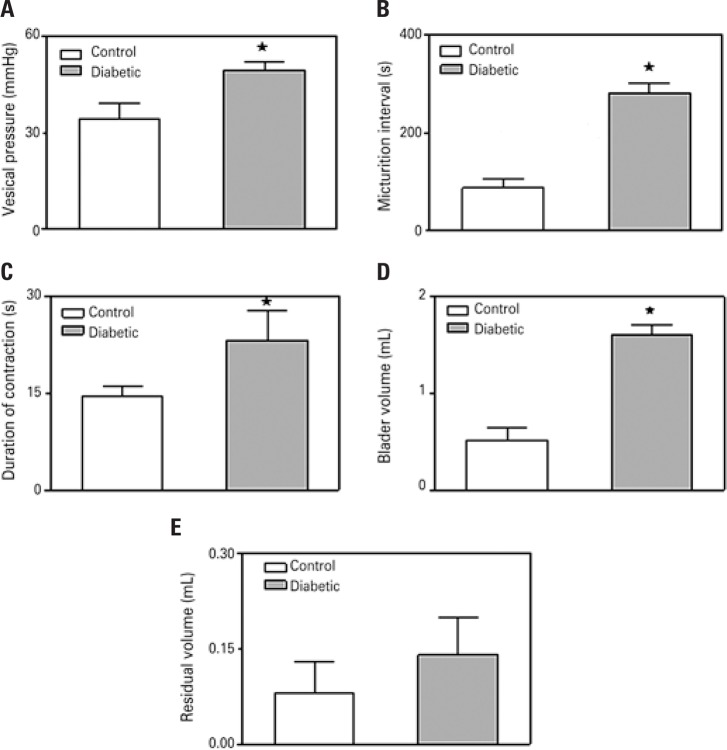
Values (mean±SEM) of intravesical pressure (A), interval (B), duration (C), micturition volume (D) and post-voiding residual volume (E) in non-anesthetized control and chronic diabetic rats.*p<0.05

In control animals continuous infusion of saline (0.2mL/min) caused periodical elevations of IVP associated with emission of urine. These elevations of IVP were considered micturition contractions. Maximal IVP during the micturition contraction was significantly greater in rats with alloxan-induced diabetes (34.6±4.7cmH_2_O *versus* 49.6±2.5cmH_2_O) (Figure 1A).

The duration of micturition contractions (14.5±1.7 seconds *versus* 23.33±4.6 seconds) as well as the interval between micturition contractions (87.5±17.02 seconds* versus* 281.11±20.24 seconds) were significantly greater in the diabetic rats (Figures 1B and 1C). The voided urine volume with each contraction was also significantly greater in diabetic rats (1.60±0.1mL *versus* 0.52±0.1mL) (Figure 1D), whereas the calculated residual volume of both groups was not significantly different (0.08±0.05mL *versus* 0.14±0.06mL) (Figure 1E).

### *In vitro *experiments

**Figure 2:**
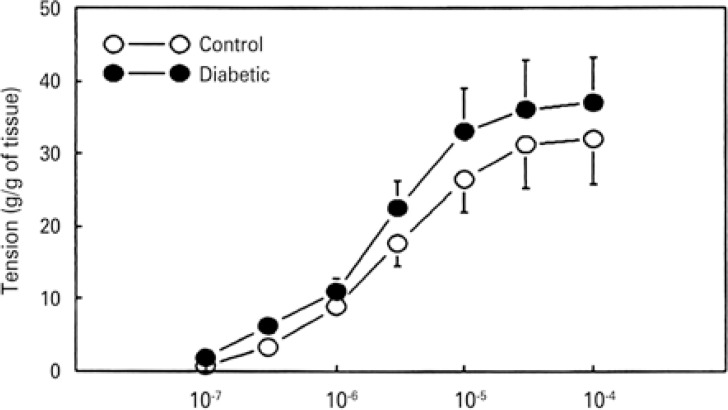
Concentration-effect curves for bethanechol in isolated bladder strips from control (n=4) and alloxan-induced diabetes rats (n=4)

In isolated bladder strips from both control and diabetic animals, bethanechol (0.1 to 100µM) caused concentration-dependent contractions with similar potency (EC_50 _3µM *versus* 5µM) and maximal effect (31.2±5.9g/g *versus* 36.1±6.8g/g) (Figure 2).

**Figure 3:**
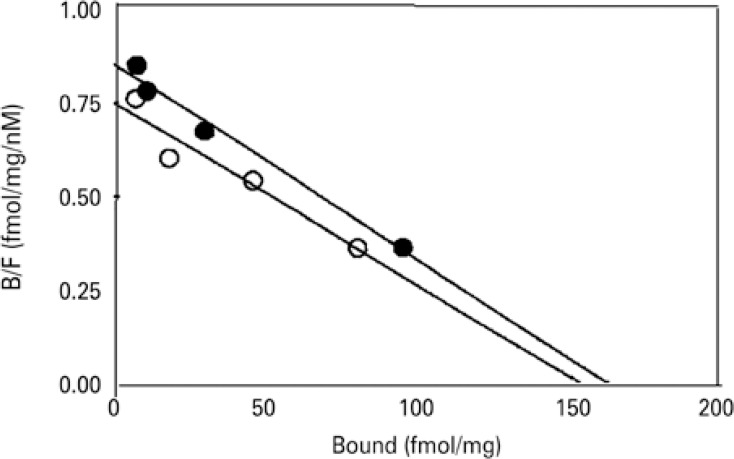
Scatchard plot analysis of specific [3H]-NMS binding sites in rat bladder homogenates control (O, n=3) and diabetic (●, n=3) rats

In addition, no differences were found in the total number (169±4fmol/mg protein *versus* 176±3fmol/mg protein) or affinity (0.69±0.1nM *versus* 0.57±0.1nM) of the specific [^3^H]-NMS binding sites present in bladder homogenates from both diabetic and control animals (Figure 3).

## DISCUSSION

The present study was conceived to re-examine urinary bladder function and muscarinic receptor functional and biochemical characteristics in alloxan-induced diabetes rats, kept with no treatment for a long period of time (11 months) to mimic human chronic diabetes. Alloxan-induced diabetes rats showed increased urinary output and urinary bladder weight which is consistent with similar observations found in several animal models of *diabetes mellitus*.^5,6,12-15,17,18^ Previous studies in alloxan-induced diabetes rats evaluated bladder function in anesthetized animals under non-voiding (isovolumetric) conditions after 4 or 6 months of alloxan administration. Considering that such conditions are rather non-physiological, one of the aims of the present study was to explore bladder function in awaken animals and under voiding conditions which are more relevant physiologically. The main cystometric findings of the present study include higher maximal IVP and duration of micturition contractions, as well as higher micturition contraction interval in diabetic rats; no differences were observed in residual volume between control and diabetic animals. In addition, and in contrast with what was observed in voiding CMG of non-anesthetized chronic STZ-induced diabetes rats,^9^ during the filling phase of CMG, no IVP variations (non-voiding contractions) were observed between micturition contractions in alloxan-induced diabetes rats for 11 months. The observation that alloxan-induced diabetes rats exhibited significant elevation of maximal micturition pressure during a conscious voiding CMG is, to the best of our knowledge, original and it was not observed in a previous study, in which voiding CMG was performed in non-anesthetized STZ-induced diabetes animals, for 6 months;^17^ in addition, no differences were observed in maximal IVP in voiding CMG in anesthetized STZ-induced diabetes rats for 2 months.^7,8^However, conscious voiding CMG studies in rats with STZ-induced diabetes showed an increase in maximal IVP in earlier stages of diabetes (6 to 9 weeks).^9,17,18^ It is worth noting that the latter study^18^ showed that in 12 and 20 weeks diabetic rats, the maximal IVP was significantly decreased. These authors proposed that urinary bladder function in chronic diabetic animal models undergo a transition from a compensated (early stages of *diabetes mellitus*), to a decompensated state. This appears not to be the case in alloxan-induced diabetes rats, since the present study showed that after 11 months of diabetes the maximal IVP was still significantly higher in the diabetic animals. Although the increased micturition pressure observed in conscious voiding CMG studies of STZ-induced diabetes rats for 8 to 9 weeks^9,18^ could be attributed to an increase in detrusor muscle sensitivity to acetylcholine.^12^ This explanation does not apply to the results of the present study, since no differences were observed in the potency or maximal effect of bethanechol to cause contractions of isolated detrusor strips. Consistent with the present observations, previous studies in chronic (5 weeks or 3 months) alloxan-induced diabetes rats showed no differences in detrusor contractions elicited by bethanechol or nerve stimulation.^6,15^ Furthermore, in chronic STZ-induced or BB diabetic rats detrusor responses to cholinergic agonists or nerve stimulation were found to be significantly diminished^7,13,14^ or unchanged.^13,17^ Although an increase in the total number of receptors in bladder domes of 2- and 4-week STZ-induced diabetes rats has been described;^12^ in the present study no differences were observed in the affinity or total number of specific [^3^H]-NMS binding sites in bladder homogenates between control and alloxan-induced diabetes rats. It is worth noting that since in our study the specific binding of NMS was corrected by the protein content of the homogenates our findings are consistent with the data presented by Latifpour et al.^12^ when corrected in the same manner. Thus, the elevated maximal micturition IVP observed in the diabetic animals is most likely the result of the bladder hypertrophy present in these animals.

The fact that the micturition contraction interval was increased in diabetic rats is an indication that the threshold volume required to trigger the micturition reflex was also increased. Indeed, considering the infusion flow and the micturition interval the estimated threshold volume increased from 0.29mL in the controls animals to 0.94mL in the diabetic animals. This difference is an indication of a sensory bladder *déficit* in long-term alloxan-induced diabetes rats and is entirely consistent with previous observations^7,8,18^ and most likely related to A-delta and C fibers abnormalities described in diabetic animals.^16,19^

## CONCLUSION

This study showed for the first time that micturition contractions during voiding cystometry in conscious long-term (11 months) alloxan-induced diabetes rats had higher amplitude and duration, which could be attributed to changes in the functional or biochemical characteristics of bladder muscarinic receptors. It showed, in addition, that the interval between micturition contractions was greatly increased in alloxan-induced diabetes rats indicating a sensory *déficit* most likely related to bladder afferent sensory-motor fiber neuropathy.
