# Supplementary material for: Functional and biochemical characteristics of urinary bladder muscarinic receptors in long-term alloxan diabetic rats
Source: Einstein (Sao Paulo). 2015 Jul-Sep;13(3):404–9. doi: 10.1590/S1679-45082015AO3233 (PMC4943786; doi:10.1590/S1679-45082015AO3233)
Supplement: Translation

# Translation: Características funcionais e bioquímicas dos receptores muscarínicos da bexiga de ratos com diabetes crônico induzido por aloxana

**Authors:** Jeová Nina Rocha

**Affiliations:** 1 Faculdade de Medicina de Ribeirão Preto, Universidade de São Paulo, Ribeirão Preto, SP, Brasil.

**Keywords:** Bexiga urinária/metabolismo, Aloxano/efeitos adversos, *Diabetes mellitus* experimental, Receptores muscarínicos, Micção/efeitos de drogas, Ratos Wistar

## Abstract

**Objetivo:**

Reestudar o funcionamento da bexiga *in vivo* e determinar as características funcionais e bioquímicas dos receptores muscarínicos vesicais de ratos com diabetes crônico induzido por aloxana.

**Métodos:**

Ratos Wistar de dois meses de idade receberam injeção de aloxana, e os animais que apresentaram glicemia >300mg/dL foram mantidos por 11 meses junto de outros não tratados e pareados por idade. Nos dois grupos de animais, peso corpóreo, peso da bexiga, glicemia e volume urinário de 24 horas foram medidos. Em ambos os grupos, realizou-se a cistometria miccional em animais não anestesiados. Foram determinados os seguintes parâmetros: pressão máxima de micção, intervalo e contração de micção, bem como o volume de esvaziamento e o volume residual pós-miccional. Além disso, foram determinadas as curvas de concentração-resposta a betanecol em preparações isoladas de bexiga e também as características dos sítios de ligação da [^3^H]-N-metil-escopolamina em homogenatos de bexiga.

**Resultados:**

O peso médio da bexiga foi de 162,5±21,2mg *versus* 290±37,9mg nos animais controles e tratados, respectivamente (p<0,05). A amplitude de contração (34,6±4,7mmHg *versus* 49,6±2,5mmHg), a duração (14,5±1,7 segundos* versus* 23,33±4,6 segundos) e o intervalo (87,5±17,02 segundos *versus* 281,11±20,24 segundos) de micção foram significantemente maiores nos ratos tratados com aloxana. O volume de urina eliminada durante a contração miccional também foi maior nos animais diabéticos. Contudo, o volume residual pós-miccional não foi estatisticamente diferente. Não foram observadas diferenças na resposta ao betanecol (EC50 3µM *versus* 5µM) e no seu efeito máximo (31,2±5,9g/g *versus* 36,1±6,8g/g) em preparações isoladas de bexiga, bem como no número total (169±43fmol/mg *versus* 176±3fmol/mg) e na afinidade (0,69±0,1nM *versus* 0,57±0,1nM) dos receptores muscarínicos da bexiga.

**Conclusão:**

O funcionamento da bexiga *in vivo* está alterado no diabetes crônico induzido por aloxana, porém sem alterações funcionais e bioquímicas nos receptores muscarínicos da bexiga.

## INTRODUÇÃO

Após o relato inicial de Jordan et al.^1^ de seis pacientes diabéticos que apresentavam paralisia da bexiga, as evidências acumuladas indicam que os pacientes diabéticos crônicos frequentemente apresentam sintomas de disfunção da bexiga.^2^ Os sintomas de disfunção diabética da bexiga, denominada cistopatia diabética, quando associada a neuropatia periférica,^3^ incluem hiporreflexia miccional, aumento da capacidade vesical, retenção urinária e até completa atonia vesical.

Os efeitos do diabetes crônico sobre o funcionamento da bexiga foram estudados em diversos modelos animais de diabetes. Embora a maioria dos estudos tenha mostrado aumento da complacência vesical e do volume limiar necessário para provocar contrações miccionais, foram descritos resultados conflitantes no tocante à função contrátil do músculo detrusor e seu controle nervoso em animais diabéticos. Estudos com cistometria (CMG) sob condições isovolumétricas em ratos *Big Blue* (BB) diabéticos de 4 e 6 meses,^4^ por exemplo, mostraram aumento no limiar de volume e na amplitude das contrações vesicais. Por outro lado, em ratos de 3 e 6 meses com diabetes induzido por aloxana não foram observadas diferenças na amplitude das contrações da bexiga sob condições isovolumétricas.^5,6^ De modo semelhante, estudos de CMG em condições miccionais, realizados em ratos anestesiados de 7 a 9 semanas com diabetes induzido por estreptozotocina (STZ), não apresentaram diferenças na amplitude das contrações miccionais.^7,8^ No entanto, um estudo de CMG em condições miccionais realizado em ratos com diabetes induzido por STZ de 8 a 11 semanas não anestesiados mostrou que a amplitude das contrações miccionais tinha aumentado muito.^9^ Por outro lado, em um estudo recente realizado em ratos diabéticos Goto-Kakizaki de 10 e 46 semanas, observou-se que a amplitude das contrações miccionais diminuiu significativamente.^10^

Também foram descritas observações conflitantes em relação à resposta contrátil do detrusor à estimulação nervosa ou a agonistas muscarínicos; por exemplo, as contrações do músculo detrusor induzidas por agonistas muscarínicos em animais diabéticos foram descritas como aumentadas,^11-13^ diminuídas^14^ ou inalteradas.^15^

## OBJETIVO

Reestudar o funcionamento da bexiga *in vivo* e determinar as características funcionais e bioquímicas dos receptores muscarínicos vesicais em ratos com diabetes de longo prazo induzido por aloxana.

## MÉTODOS

Os procedimentos experimentais foram aprovados pelo Comitê de Ética Animal da Faculdade de Medicina de Ribeirão Preto da Universidade de São Paulo (Protocolo n^o^ 051/2011.) Todos os experimentos foram realizados no Laboratório de Neuro-Urologia da Faculdade de Medicina de Ribeirão Preto.

Foi induzido diabetes em ratos machos Wistar de 2 meses por meio de uma única injeção intravenosa de aloxana (40mg/kg, diluído em solução fisiológica a 0,9%); 2 dias após a administração de aloxana, na ausência de jejum, foram colhidas amostras sanguíneas da cauda para determinação da glicemia em sangue total (glicosímetro Beckman, Brea, Califórnia, Estados Unidos). Os animais com glicemia ≥300mg/dL foram considerados diabéticos e incluídos no estudo. Ratos não tratados pareados por idade foram incluídos como controle. Ambos os grupos de animais foram mantidos por 11 meses em condições similares de alojamento (temperatura ambiente de 22±2°C, ciclo claro-escuro de 12/12 horas), com acesso *ad libitum* a água e comida.

Onze meses após a injeção de aloxana, na ausência de jejum, foram colhidas amostras sanguíneas da cauda de ambos os grupos, para determinação da glicemia em sangue total. Em seguida, os animais controle e diabéticos foram colocados em gaiolas metabólicas para determinar o débito urinário de 24 horas. As gaiolas metabólicas tinham uma rede especial que permitia que a urina fosse separada das fezes. Após o estudo na gaiola metabólica, quatro ratos controle e cinco ratos diabéticos foram anestesiados com éter sulfúrico, submetidos a tricotomia da parede abdominal ventral e antissepsia com betadine, com exposição da bexiga via incisão abdominal na linha média inferior. Uma das pontas do cateter de polietileno (PE-50) foi submetida a uma temperatura elevada para formar um pequeno colar; esse cateter foi implantado no ápice da bexiga e fixado com uma sutura. A outra ponta do cateter foi tunelizada por via subcutânea e exteriorizada na região lombar do animal. Após o procedimento, o cateter foi conectado a uma torneira de três vias, que estava conectada a um transdutor de pressão, posicionado no nível da sínfise púbica do animal. A solução fisiológica de salina foi infundida. Em seguida, o animal foi colocado em uma gaiola especial de contenção para coletar urina eliminada a cada contração miccional. CMG miccional contínua foi realizada após os ratos se recuperarem completamente da anestesia. Para esse procedimento, um cateter vesical foi conectado por meio de uma torneira de três vias a um transdutor de pressão (World Precision Instruments Inc., Sarasota, Estados Unidos) e a uma bomba de infusão (Bomba de Infusão KD Scientific, Modelo 780.200, número de série 201755, Estados Unidos). O transdutor de pressão foi posicionado ao nível do osso púbico do animal, infundindo-se solução salina (em temperatura ambiente) de modo contínuo (velocidade de infusão 0,2mL/min) para induzir as contrações miccionais. A pressão intravesical (PIV) foi registrada continuamente em papel térmico utilizando-se um polígrafo multicanal (Narco BioSystems MK III, Houston, Texas, Estados Unidos). Após um período de equilíbrio de 20 a 30 minutos, uma CMG de 30 minutos foi registrada para análise. Foram analisados os seguintes parâmetros da CMG: a pressão miccional máxima (PIV desenvolvida durante as contrações miccionais); intervalo entre as micções (intervalo de tempo entre o retorno da PIV ao valor inicial, após uma contração miccional e o início da contração miccional seguinte, ou seja, o ponto no tempo em que a PIV apresentou uma rápida elevação); e duração das contrações miccionais, tempo decorrido desde o início da contração miccional (ver acima) até o retorno da PIV à linha de base. Após registrar a CMG por 30 minutos, a infusão foi interrompida, e a bexiga, esvaziada. A infusão foi, então, reiniciada, prosseguindo até o início da emissão de urina que acompanhava a primeira contração miccional. O volume de urina eliminado foi, então, medido e usado para calcular o volume residual pós-miccional por meio da seguinte fórmula: volume de solução salina infundido menos volume eliminado.

### Experimentos *in vitro*

Após o estudo na gaiola metabólica, outros quatro ratos controle e quatro ratos diabéticos foram anestesiados com éter sulfúrico e submetidos à exposição e remoção da bexiga por meio de incisão abdominal na linha média inferior. As bexigas foram colocadas numa placa de Petri contendo uma solução de Krebs modificada (em mM: NaCl 119,4; NaHCO_3_ 25,00; KCl 4,7; MgCl_2 _1,31; CaCl_2 _2,5; KH_2_PO_4 _1,17; glicose 11,1) e, após remoção dos tecidos aderentes longitudinais, uma tira de bexiga (15mm de comprimento x 3mm de largura) foi montada para registro das contrações isométricas num banho de órgãos de 10mL, contendo a mesma solução de Krebs mantida a 37°C e continuamente borbulhada com O_2_ a 95%/CO_2_ a 5%. A tira de músculo detrusor, sob tensão basal de 1g, foi deixada para estabilizar por 60 minutos, com troca da solução de Krebs a cada 15 minutos. Após esse período de equilíbrio, foram preparadas curvas concentração-resposta cumulativa para o agonista muscarínico betanecol. Depois do experimento, as tiras de bexiga foram removidas, secas em papel filtro e pesadas. O peso das tiras foi usado para normalizar a força contrátil.

### Determinações bioquímicas

Depois do estudo na gaiola metabólica, seis animais, três controle e três diabéticos, foram anestesiados com éter sulfúrico, sendo removida a bexiga deles, que foi cortada em pequenos fragmentos e imediatamente homogeneizada em HEPES Na^+^/Mg^+^ gelado, pH 7,4 (NaCl=100mM, MgCl_2_=10mM e HEPES=20mM) utilizando-se um homogeneizador Brinkman Polytron em velocidade 6 (12 impulsos de 5 segundos a intervalos de 15 segundos). O homogenato foi filtrado em malha de *nylon* e centrifugado a 13.000rpm por 20 minutos. A fração do sobrenadante foi descartada e o *pellet *foi novamente dissolvido em tampão HEPES e diluído a uma concentração proteica final de 1,0mg/m. A concentração proteica foi determinada pelo método de Lowry et al.,^16^ usando albumina de soro bovino como padrão. Amostras duplicadas de 0,5mL do homogenato (correspondentes a 0,5mg de proteína) foram incubadas a 32°C em agitador Dubnoff por 45 minutos, com diversas concentrações de [^3^H]-N-metil-escopolamina ([^3^H]-NMS, 0,1 a 10nM) na ausência e na presença de um excesso de cem vezes de escopolamina não marcada. A incubação foi encerrada pela adição de tampão de HEPES gelado e filtração rápida sob vácuo por meio de filtros de fibra de vidro GF/B. Os filtros foram enxaguados duas vezes com tampão HEPES gelado e transferidos para frascos de cintilação contendo 8mL de Aquasol (NEN, Boston, Massachusetts, Estados Unidos). Após vigorosa agitação, os filtros foram deixados por 24 horas no escuro para extrair a radioatividade e contados num contador de cintilação em meio líquido (Packard 1500, Califórnia, Estados Unidos) por 2 minutos. A ligação específica foi definida como a diferença de radioatividade na presença e na ausência de escopolamina para cada concentração de ^[3H^. Foram calculados os valores de Kd e Bmax da ligação específica, expressos em mg de proteína, usando-se o método de regressão linear de Scatchard.

### Análise estatística

Todos os valores dos parâmetros da CMG e da tensão máxima das tiras de bexiga foram expressos como média±SEM. A concentração do betanecol necessária para causar contrações de magnitude igual a 50% da máxima (EC_50_) para cada tira foi calculada como a média geométrica. A significância estatística das diferenças entre os ratos controle e os diabéticos foi determinada usando-se o teste *t* de Student. Um valor de p<0,05 foi considerado estatisticamente significativo.

## RESULTADOS

Os níveis de glicemia nos animais com diabetes induzido por aloxana foram significativamente maiores do que nos animais controle (p<0,05) (Tabela 1). Ambos os grupos de animais apresentaram aumento no peso corpóreo durante o período de 11 meses. No entanto, os ratos com diabetes induzido por aloxana ganharam significativamente menos peso. Os ratos diabéticos também apresentaram peso da bexiga e débito urinário de 24 horas significativamente maiores (Tabela 1).

**Tabela 1:** Efeito do *diabetes mellitus* induzido por aloxana no peso corpóreo do rato, peso da bexiga, concentração de glicose no sangue e diurese de 24 horas

	Controles (n=8)	Diabéticos (n=9)
Peso corporal, g		
Inicial	150,0±5,0	160,0±20,0
Final	520,0±30,8	330,0±10,0*
Peso da bexiga, mg	162,5±21,2	290,0±37,9*
Glicemia, mg/dL	118,0±17,1	536,0±25,9*
Diurese, mL/24 horas	20,2±3,7	103,0±7,85*

*Estatisticamente diferente do controle (p<0,05).

### Cistometria miccional contínua

Nos animais controle, a infusão contínua de solução salina (0,2mL/min) causou elevações periódicas da PIV associadas à emissão de urina. Elevações da PIV foram consideradas contrações miccionais. A PIV máxima durante a contração miccional foi significativamente maior nos ratos com diabetes induzido por aloxana (34,6±4,7cmH_2_O *versus* 49,6±2,5cmH_2_O) (Figura 1A).

**Figura 1:** Valores (média±SEM) da pressão intravesical (A), intervalo (B), duração (C), volume de micção (D) e volume residual pós-miccional (E) em ratos não anestesiados (ratos controle e diabéticos). *p<0,05

A duração das contrações miccionais (14,5±1,7 segundos* versus* 23,33±4,6 segundos) bem como o intervalo entre as contrações miccionais (87,5±17,02 segundos *versus* 281,11±20,24 segundos) foram significativamente maiores nos ratos diabéticos (Figuras 1B e 1C). O volume urinário eliminado a cada contração também foi significativamente maior nos ratos diabéticos (1,60±0,1mL *versus* 0,52±0,1mL) (Figura 1D), embora o volume residual calculado de ambos os grupos não tenha sido significativamente diferente (0,08±0,05mL *versus* 0,14±0,06mL) (Figura 1E).

### Experimentos *in vitro*

Nas tiras de bexiga isoladas tanto de animais controle quanto diabéticos, o betanecol (0,1 a 100µM) causou contrações dependentes de concentração com potência (EC_50 _3µM *versus* 5µM) e efeito máximo (31,2±5,9g/g *versus* 36,1±6,8g/g) semelhantes (Figura 2).

**Figura 2:** Curvas de concentração-resposta para o betanecol em tiras de bexiga isoladas de ratos controle (n=4) e ratos com diabetes induzido por aloxana (n=4)

Além disso, não foram observadas diferenças no número total (169±4fmol/mg de proteína* versus* 176±3fmol/mg de proteína) ou na afinidade (0,69±0,1nM *versus* 0,57±0,1nM) dos sítios de ligação específica da [^3^H]-NMS nos homogenatos, tanto de animais controle quanto nos diabéticos (Figura 3).

**Figura 3:** A análise de Scatchard de sítios de ligação específica do [3H]-NMS em homogenatos de bexiga de ratos controle (O, n=3) e diabéticos (●, n=3)

## DISCUSSÃO

O presente estudo foi concebido para rever as funções vesicais e as características funcionais e bioquímicas dos receptores muscarínicos em ratos com diabetes induzido por aloxana, mantidos sem tratamento por um longo período de tempo (11 meses), de modo a mimetizar o diabetes crônico humano. Os ratos com diabetes induzido por aloxana apresentaram maior débito urinário e peso da bexiga, de modo condizente com observações semelhantes encontradas em vários modelos animais de *diabetes mellitus*.^5,6,12-15,17,18^ Estudos anteriores com ratos com diabetes induzido por aloxana avaliaram a função vesical em animais anestesiados sob condições não miccionais (isovolumétricas), após a administração de aloxana por 4 ou 6 meses. Considerando que essas condições não são fisiológicas, um dos objetivos do presente estudo foi estudar a função vesical em animais despertos e sob condições miccionais mais relevantes em termos fisiológicos. Os principais achados cistométricos do presente estudo incluem maior PIV máxima e duração das contrações miccionais, bem como maior intervalo de contração miccional nos ratos diabéticos; nenhuma diferença foi observada no volume residual, comparando-se os ratos do grupo controle com o dos animais diabéticos. Além disso, ao contrário do que foi observado em CMG miccional de ratos com diabetes crônico induzido por STZ,^9^ durante a fase de enchimento da CMG, não foram observadas variações da PIV entre as contrações miccionais nos ratos com diabetes induzidos por aloxana por 11 meses. A observação de que os ratos com diabetes induzido por aloxana apresentaram uma elevação significativa da pressão máxima de micção durante uma CMG miccional consciente é, pelo que se sabe, inédita e não observada em um estudo prévio, em que a CMG miccional tenha sido realizada em animais com diabetes induzido por STZ, por 6 meses.^17^ Além disso, nenhuma diferença foi observada na PIV máxima na CMG miccional em ratos anestesiados com diabetes induzido por STZ por 2 meses.^7,8^ Contudo, os estudos de CMG miccionais em ratos conscientes com diabetes induzido por STZ mostraram um aumento na PIV máxima nos estágios iniciais do diabetes (6 a 9 semanas).^9,17,18^ É importante salientar que esse último estudo^18^ mostrou que em ratos diabéticos de 12 e 20 semanas, a PIV máxima estava significativamente diminuída. Esses autores propuseram que a função da bexiga nos modelos animais de diabetes crônico passa por uma transição de um estado compensado (estágios iniciais do *diabetesmellitus*) para um estado descompensado. Esse não parece ser o caso nos ratos com diabetes induzido por aloxana, uma vez que o presente estudo mostrou que, após 11 meses de diabetes, a PIV máxima ainda era significativamente mais elevada nos animais diabéticos, embora a pressão miccional aumentada observada nos estudos de CMG miccionais conscientes em ratos com diabetes induzido por STZ por 8 a 9 semanas^9,18^ pudesse ser atribuída a uma sensibilidade aumentada do músculo detrusor à acetilcolina.^12^ Essa explicação não se aplica aos resultados do presente estudo, uma vez que não foram observadas diferenças na potência ou no efeito máximo do betanecol em causar contrações em tiras isoladas de músculo detrusor. De modo condizente com essas observações, estudos anteriores realizados em ratos com diabetes crônico (5 semanas ou 3 meses) induzido por aloxana não mostraram diferença nas contrações do músculo detrusor induzidas por betanecol ou estimulação nervosa.^6,15^ Além disso, em ratos com diabetes crônico induzido por STZ ou em ratos BB diabéticos, verificou-se que as respostas do músculo detrusor a agonistas colinérgicos ou a estimulação nervosa estavam significativamente diminuídas^7,13,14^ ou inalteradas.^13,17^ Embora tenha sido descrito um aumento no número total de receptores do ápice da bexiga de ratos de 2 e 4 semanas com diabetes induzido por STZ,^12^ no presente estudo não foram observadas diferenças na afinidade ou no número total de sítios específicos de ligação [^3^H]-NMS nos homogenatos de bexiga, ao compararmos os ratos controle com os ratos com diabetes induzido por aloxana. Vale notar que como em nosso estudo a ligação específica de NMS foi corrigida pelo conteúdo proteico dos homogenatos, nossos achados são consistentes com os dados apresentados por Latifpour et al.,^12^ quando corrigidos do mesmo modo. Portanto, a PIV miccional máxima elevada observada nos animais diabéticos é muito provavelmente decorrente da hipertrofia da bexiga presente nesses animais.

O fato de o intervalo entre as contrações miccionais estar elevado nos ratos diabéticos é indicativo de que o limiar de volume exigido para desencadear o reflexo miccional também estivesse aumentado. De fato, considerando o fluxo de infusão e o intervalo miccional, o limiar de volume estimado aumentou de 0,29mL nos animais controle para 0,94mL nos animais diabéticos. Essa diferença indica um *déficit *sensitivo vesical nos ratos com diabetes de longo prazo induzido por aloxana, sendo bem condizente com observações prévias^7,8,18^ e muito provavelmente estando relacionada a anormalidades nas fibras A-delta e C descritas nos animais diabéticos.^16,19^

## CONCLUSÃO

Este estudo mostrou pela primeira vez que as contrações miccionais durante a cistometria em ratos conscientes com diabetes de longo prazo (11 meses) induzido por aloxana tiveram maior amplitude e duração, que não puderam ser atribuídas a mudanças nas características funcionais ou bioquímicas dos receptores muscarínicos da bexiga. Ele mostrou, além disso, que o intervalo entre as contrações miccionais estava muito aumentado nos ratos com diabetes induzido por aloxana, indicando *déficit* sensitivo muito provavelmente relacionado à neuropatia de fibras sensitivo-motoras aferentes da bexiga.
